# Reactive Arthritis due to *Shigella* Infection after a Visit to Egypt: A Late Complication of an Intestinal Infection

**DOI:** 10.1155/2012/972517

**Published:** 2012-09-06

**Authors:** Alwin Tilanus, Rembert Mertens, Lucie Seyler, Patrick Lacor, Brigitte Velkeniers

**Affiliations:** Department of Internal Medicine/Infectious Diseases, University Hospital Brussels, Laarbeeklaan 101, 1090 Brussels, Belgium

## Abstract

We describe a case of reactive arthritis following *Shigella* infection after a trip to Egypt. The diagnostic challenge and treatment of this acute medical condition are discussed.

## 1. Introduction

Reactive arthritis is by definition a aseptic arthritis that can be caused by various infectious agents. Among these agents, the *Shigella* species are frequently identified. After a initial period of dysentery, a rheumatoid disorder might appear in which various organ system can be affected in varying degrees of severity.

Arthritis, conjunctivitis, and urethritis (a triade formerly known as “Reiter syndrome”) is a well-known clinical presentation, but these symptoms do not have to occur necessarily at the same time [1].

Diagnosis can be challenging and complicated as we will try to show in this case. This paper serves to alert the clinician to the possible widespread consequences of reactive arthritis.

## 2. Case Report

A 24-year-old male presented initially to the emergency room with complaints of red and swollen eyes after a period of about one week of (nonbloody) diarrhea that started approximately three weeks before. He was discharged initially with the diagnosis of conjunctivitis (not confirmed by a ophthalmologist). He returned two weeks later with a painful swollen right knee accompanied by fever, general malaise, and back pain. The patient had been in Egypt 6 weeks earlier for one week. In Egypt, he had only been to the sea for diving. There was no history of insect bites. He had only ridden a camel and there had been no contact with other animals. There was no history of (unsafe) sexual contact. The patient informs us that he had travelled back via Poland and that he had eaten a typical sausage. The patient's medical history was blank. He started taking medication against diarrhea (paromomycine) and some acetaminophen.

On physical examination, we saw a non-ill-appearing patient; blood pressure: 102/59 mm Hg; pulse: 82/min, regular temperature: 36.3°C. The skin showed no abnormalities. Ophthalmological examination revealed signs of bilateral conjunctivitis. No palpable lymph nodes were found in the cervical or inguinal region. Chest auscultation revealed normal heart and breath sounds. Examination of the abdomen revealed normal peristalsis and slight tenderness on palpation without guarding and hepatosplenomegaly. The right knee was red, swollen, and painful with mobilization.

Laboratory examination revealed an elevated and rapidly increasing CRP (C-reactive protein), ESR (Erythrocyte sedimentation rate), leukocytosis with elevated neutrophils (no eosinophilia) with a limited normocytic anemia. Furthermore, there were elevated liver enzymes (liver transaminases as well as alkaline phosphatase and gamma-glutamyltransferase). Antinuclear antibodies were negative. Serologic tests for *Entamoeba histolytica*, several helmintic infections, *Yersinia*, *Campylobacter*, and *Borrelia burgdorferi* remained negative.

Examination of the urine revealed no abnormalities. A chest X-ray was normal. A puncture of the right knee was performed and while awaiting the culture result the patient was treated with flucloxacillin with an initial poor clinical response. Therefore, arthroscopy and joint flushing was performed. The punctate revealed many leukocytes but no bacteria. Hemocultures remained negative.

**Figure 1:**
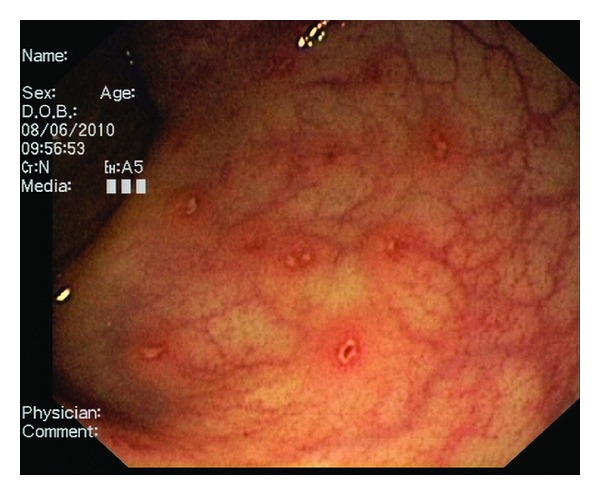
Colonoscopy of the patient showed an aspecific colitis, with clear ulcerations.

A CT scan of the abdomen showed a liver cyst, a thickened wall of the colon descendens/ascendens indicating colitis, para-aortic lymphadenopathy, and free fluid in the Douglas Space. A subsequent left colonoscopy showed colitis with aphthoid ulcerations (Figure 1). Biopsy showed aspecific colitis (no inflammatory bowel disease).

Finally, one feces culture revealed *Shigella* species and confirmed initial suspicion of reactive arthritis. After a week, the patient made a good recovery and was discharged with diclofenac 75 mg 2x/d as necessary.

## 3. Discussion

We describe a case of reactive arthritis following *Shigella* infection.

Gram negative *Shigella* bacteria (rod shaped, nonsporulating) belong to the family Enterobacteriaceae. Four species of *Shigella* (*S. dysenteriae, S. flexneri, S. boydii, and S. sonnei*) have been identified. *Shigella* bacteria are transmitted by the orofecal route and incidence correlates with areas of poor water hygiene. After infection, *Shigella* bacteria indirectly enter colonic epithelial cells via microfold cells (M-cells) where they first encounter macrophages. Breakdown is avoided by causing apoptosis of macrophages. Subsequently, the bacteria multiply and spread laterally to infect adjacent epithelial cells. This process activates proinflammatory signaling pathways and finally NK cells and polymorphonuclear mononuclear cells (PMNs) that eliminate the bacteria but cause mucosal ulceration, inflammation, and bleeding. Symptoms of Shigellosis include (mucoid bloody) diarrhea, abdominal cramps, and tenesmus [2, 3].

Colonoscopy of our patient showed signs of an aspecific colitis with aphthoid ulcerations and stool culture enabled diagnosis of *Shigella flexneri* infection.

Reactive arthritis (ReA) is defined as an (aseptic) inflammatory arthritis that belongs to the family of seronegative spondyloarthritis. When the arthritis presents with (nongonococcal) urethritis and conjunctivitis, it was traditionally known as “Reiter syndrome,” though the term has been largely replaced by the term reactive arthritis. ReA can be triggered by infectious diarrhea caused by bacteria such as *Salmonella*, *Shigella*, *Campylobacter*, and *Yersinia*, but other pathogens have also been associated with ReA especially *Chlamydia* [1, 4].

The classic triad of arthritis, conjunctivitis, and urethritis however is present only in a minority of patients. Therefore, because of overreliance on this classic triad of symptoms incidence numbers of ReA may be well underestimated [1, 5].

In a study from Finland, a questionnaire was sent to 278 patients with a *Shigella* positive stool to examine whether they had signs of ReA. The examiners concluded that ReA occurred in as much as 7% of patients after *Shigella* infection, with *S. Sonnei* being the most frequent pathogen. Furthermore, 36% of the patients was HLA-B27+ [6].

An acute reactive syndrome typically starts within 1 to 4 weeks after the initial infection followed by gradual resolution of symptoms which can take sometimes up to months or years. Symptoms can be diverse including articular (usually oligoarthritis or tendonitis/enthesitis, mucosal, cutaneous (in particular keratoderma blennorrhagica), ocular (mainly conjunctivitis) with or without systemic features (fever, malaise, and weight loss). Systemic symptoms usually occur during the acute initial phase of the disease [1, 4].

Although the term reactive arthritis implicates culture negative synovia, at least bacterial products of the implicated bacteria have been demonstrated in the synovial tissue or fluid of ReA patients [1, 7].

In our case, the synovium culture was negative with elevated leukocyte counts although we did not perform PCR. Interestingly, HLA-B27 was negative.

Diagnosis of ReA is made on clinical/biochemical criteria and by exclusion of other forms of arthritis. The first criterion is having mono- or oligo-arthritis of the lower extremities. Subsequently, a puncture with culture of the synovial fluid should be performed. Laboratory investigations including autoimmune parameters (ANA, reumafactor) will safely exclude autoimmune arthritis. Stool culture, examination for *Chlamydia*, and a HIV test are considered to be the most important microbiology tests. Imaging has a minor role in diagnosis. If in doubt, an X-ray or MRI can be performed [1, 8].

In our case, mainly because of massive inflammation in the blood, a CT scan of the abdomen was performed showing signs of colitis, peritoneal fluid, and lymphadenopathy which were probably all related to the colitis and immune responses.

The pathophysiology of ReA is not fully understood and probably multifactorial. Causative organisms of ReA are incorporated into PMNs by which they are taken from the site of initial infection to the synovium of a joint where they enter synovial cells [1, 7].

HLA-B27 molecules are implicated both directly and indirectly in the pathophysiology of ReA. It is able to present (“arthritogenic”) microbial peptides to T cells, triggering an autoimmune response (“molecular mimicry”). However, the B27 molecule itself may directly serve as the autoantigen that is targeted by the immune system. In addition, Toll-like receptors (TLRs) (such as TLR-4) are able to recognize extracellular pathogens (e.g., lipopolysaccharide (LPS)) and activate immune cell responses via complex signaling pathways. Finally, an imbalance in Th1/2 cytokine profiles has been implicated in its pathophysiology [1, 7].

Treatment consists primarily of nonsteroidal anti-inflammatory drugs with good results. Since ReA by definition is reactive, there is basically no rational for antibiotics; although while awaiting culture results, treatment with an antibiotic such as penicillin or flucloxacillin seems reasonable. Second-line therapy can be local steroid injections. Disease-modifying antirheumatic drugs (DMARDs) (e.g., sulfasalazine and methotrexate) and finally TNF-alfa inhibitors (etanercept) can be reserved for nonresponders and chronic disease [1, 4, 8].
